# Experimental Evaluation of EMKEY: An Assistive Technology for People with Upper Limb Disabilities

**DOI:** 10.3390/s23084049

**Published:** 2023-04-17

**Authors:** Mireya Zapata, Kevin Valencia-Aragón, Carlos Ramos-Galarza

**Affiliations:** 1 Centro de Investigación en Mecatrónica y Sistemas Interactivos-MIST, Universidad Indoamérica, Av. Machala y Sabanilla, Quito 170103, Ecuador; kg.va1234@gmail.com (K.V.-A.); carlosramos@uti.edu.ec (C.R.-G.); 2 Facultad de Psicología, Pontificia Universidad Católica del Ecuador, Av. 12 de Octubre y Roca, Quito 170143, Ecuador

**Keywords:** assistive technology, hands-free computer interface, usability, face recognition, speech-to-text, nose tracking

## Abstract

Assistive technology can help people with disabilities to use computers more effectively and can enable them to access the same information and resources as people without disabilities. To obtain more insight into the factors that can bring about the design of an Emulator of Mouse and Keyboard (EMKEY) to higher levels of user satisfaction, an experimental study was conducted in order to analyse its effectiveness and efficiency. The experimental study involved 27 participants (*M_age_* = 20.81, *SD* = 1.14) who performed three experimental games under different conditions (using the mouse and using EMKEY with head movements and voice commands). According to the results, the use of EMKEY allowed for the successful performance of tasks such as matching stimuli (*F_(2,78)_* = 2.39, *p* = 0.10, *η*^2^ = 0.06). However, the execution times of a task were found to be higher when using the emulator to drag an object on the screen (*t_(52,1)_* = −18.45, *p* ≤ 0.001, *d* = 9.60). These results indicate the effectiveness of technological development for people with upper limb disabilities; however, there is room for improvement in terms of efficiency. The findings are discussed in relation to previous research and are based on future studies aimed at improving the operation of the EMKEY emulator.

## 1. Introduction

Assistive technologies (ATs) are a research field that is transforming the lives of impaired people [1]. These technologies are intended to provide support and assistance to people with a variety of disabilities, including mobility [2,3], vision [4] and hearing impairments [5], and cognitive disabilities [6]. It includes a variety of hardware and software approaches that apply the latest advances in technology, such as: speech recognition [7,8], computer vision [9], brain–computer interfaces [10], eye tracking [11], head and face tracking [12,13,14,15], etc. The purpose of assistive technology is to facilitate the performance of tasks that would otherwise be difficult or impossible for individuals with disabilities. In this sense, people with upper limb disabilities, which can be caused through amputation, injury, or neurological disorders [16], face unique difficulties in performing daily tasks that require the use of their hands or arms [2]. This leads to the loss of functional independence. Devices or applications for supporting people with upper limb disabilities can help them to live more independently, participate more fully in society and improve their overall quality of life [17].

In this context, the adoption of AT might vary based on variables such as the user’s unique needs and preferences, where it is important to consider how well the device meets the user’s needs and is designed according to their abilities [16]. Accessibility is also critical. It should be user-friendly, reliable and compatible with other equipment and software. Cost is another key element that can influence the acceptance of AT. Numerous individuals with impairments have low financial means; therefore, the device’s price may be a barrier to acceptance. Another important issue is the provision of proper training and continuing support for the successful use of assistive technology. Users must understand how to use the gadget effectively and how to resolve typical problems [18]. In general, the acceptability of AT has increased throughout time as more people have become aware of its benefits and as advances in technology are becoming more accessible and affordable.

In a similar vein, Human–Computer Interaction (HCI) is a discipline that concentrates on the design and assessment of interactive computing systems for human use [19,20]. The study of how people use computers, software and other digital devices, as well as how these systems can be made to better serve human requirements and tasks, are all included in this field [21] HCI focuses on developing technology that is user-friendly, straightforward and adaptable to a variety of users [22]. In this instance, HCI and assistive technology are closely related, given that HCI principles such as user-centred design, usability testing and iterative design are used in the design and evaluation of assistive technology. In this case, usability testing is a key factor in evaluating assistive technology analysing factors such as learnability, effectiveness, efficiency and user experience [21,23,24,25,26] to obtain a system that meets the needs of the end user.

AT for upper limb impairments encompasses a variety of gadgets and instruments that are developed to meet the specific needs of individuals with varying types of impairments. Each of these technologies are intended to assist individuals with upper limb disabilities in completing tasks with which they might otherwise struggle, such as eating, writing and computer use [27,28]. As a technological tool, computers are essential for society to keep people connected with each other, entertained, educated and able to work remotely. These devices need to be operated by hand. However, they cannot be used by people with partial or no mobility in their upper limbs. To overcome these problems, several systems have been developed with the aim of enabling people to access and to use a computer independently without needing assistance from others [10,11,12,13,14,29,30,31,32,33,34,35,36,37,38].

Computer vision has been used to interact with a personal computer (PC) in many ways, with the common goal of simulating the functionality of a mouse, since it is the main tool used for controlling the computer. The authors in [11,12,29,30,31] accomplished this task through eye tracking using sophisticated devices with which the user can interact. However, the use of external devices on the body can be intrusive and uncomfortable for the user. Another way to achieve mouse control is facial recognition [13,14,32,33]. In this case, landmarks are detected on the face that describe the different zones (e.g., nose, mouth and eyes) associated with cursor movement. However, these systems are developed to work only on specific applications, such as WhatsApp Web, Gmail, or Facebook, limiting their applicability. In other studies [34,35,36,37,38], the same technique has been applied and complemented with teeth recognition, a particular facial gesture, or voice commands to generate other PC functions, such as a mouse click event.

The authors in [39] present a human–machine interface for people with motor disabilities, called EMKEY (Emulator of Mouse and Keyboard). This system allows for controlling a computer using head movements and voice commands. Unlike most of the work shown previously, EMKEY is an interface that can operate without the need for an internet connection, and its use is general-purpose. This system is intended to be used without the need to acquire expensive external devices that can be intrusive. This interface has implemented several functionalities that make it more complete and useful, such as screen segmentation commands, dictation mode and an extensive list of commands that allow for the emulation of the functionality of the mouse.

EMKEY is designed for people with motor impairments who cannot use their upper limbs. The present study aims to test this interface under different conditions in order to calibrate it and to test its usability, valorating the effectiveness and efficiency. For this purpose, three games were developed and tested by 27 users using the emulator. As a first step, we worked with non-disabled users because their feedback can help to improve the system’s features. It will help with future experiments when testing the system with disabled people, giving them a better user experience. Measures such as time to win and number of failures and successes were statistically analysed to compare the different features of using EMKEY and using a regular mouse.

### EMKEY Overview

This system has two main functionalities: facial landmark recognition and the speech-to-text function. With these two features, the entire system fulfils its main task, which is to emulate the functionalities of the mouse and some keyboard commands. The Vosk library [40] was used to carry out the speech-to-text function, which controls voice command execution and dictation mode. Vosk allows for performing these tasks with good results, without an internet connection. With this function, the system listens to what the user says and compares it with a predefined list of commands. These commands allow the user to emulate common mouse events, such as clicking or double-clicking.

EMKEY also has a special feature that allows the user to move the cursor around the screen quickly through 12 voice segmentation commands. These divide the screen into 12 quadrants, and when the emulator detects that the user has spoken one of these voice commands, the cursor moves to the corresponding quadrant on the screen.

**Figure 1:**
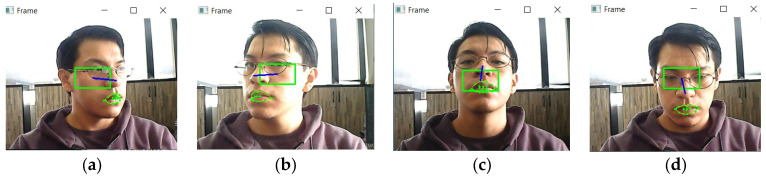
EMKEY noise detection for generating cursor movement to (**a**) right, (**b**) left, (**c**) up and (**d**) down.

On the other hand, face landmark recognition is achieved by using the OpenCV library [41] and pre-trained Dlib [42] models for face detection and facial landmark prediction. First, video signals are captured from the computer’s camera to process the frames, which are then subjected to a face detection algorithm. If a face is detected, a face mark predictor is applied. This process captures the midpoint of the nose and all mouth contours. The nose is the main control of the emulator, so when the software detects an open mouth, the central point of the nose is captured. This point is saved and a green boundary box is generated around it as shown in Figure 1. The cursor movement is generated by comparing the nose centre with the position of the green rectangle. If the user’s nose is outside the rectangle, the cursor will move towards the nearby segment. The direction of the nose movement relative to the centre of the green boundary box is represented by a blue line. This means that the cursor can move right, left, up and down, as shown in Figure 1. If the nose is inside the rectangle, the cursor will not move.

**Figure 2:**
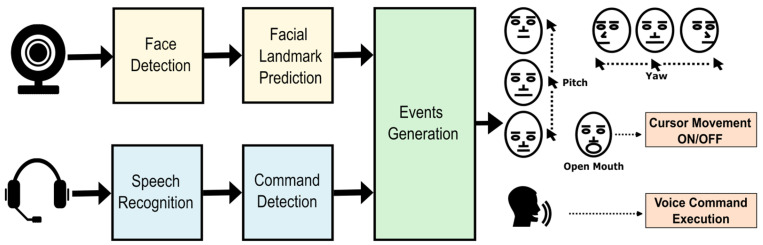
EMKEY block diagram.

Figure 2 summarises how the interface works. First, in the face recognition module, video signals are captured and then a face detector and a face landmark predictor algorithm are applied. The emulator detects the user’s open mouth to generate an on/off control for cursor movement. Pitch and yaw head movements allow the user to move the cursor up, down, right and left. Second, in parallel with the face recognition module, voice signals are processed for speech-to-text conversion, which is compared with a list of commands. If there is a match between what the user has said and the list of commands, the appropriate action is performed.

The rest of this paper is organised as follows. Section 2 describes the methods adopted in this study. Section 3 presents the results and Section 4 discusses them. Finally, Section 5 concludes the study.

## 2. Method

### 2.1. Game Experiments

Three games were developed to test the functionality of EMKEY. Each game had a different objective; thus, the EMKEY features were used in different ways. The parameters of each game were calibrated according to EMKEY’s features.

The games were developed with Scratch, a visual programming language that provides several resources for creating various media manipulations (e.g., images, sounds, music, motion and sensing) [43,44,45]. Block-based programming is accomplished by snapping together command blocks that can be created graphically using a drag-and-drop procedure without considering any syntax. The command blocks are similar to the statements of a text-based programming language. Trigger blocks link events (such as application launches, mouse clicks and key presses) to the stacks that manage these events [46]. These features were used to achieve the following applications.

#### 2.1.1. Pairing Cards Game

**Figure 3:**
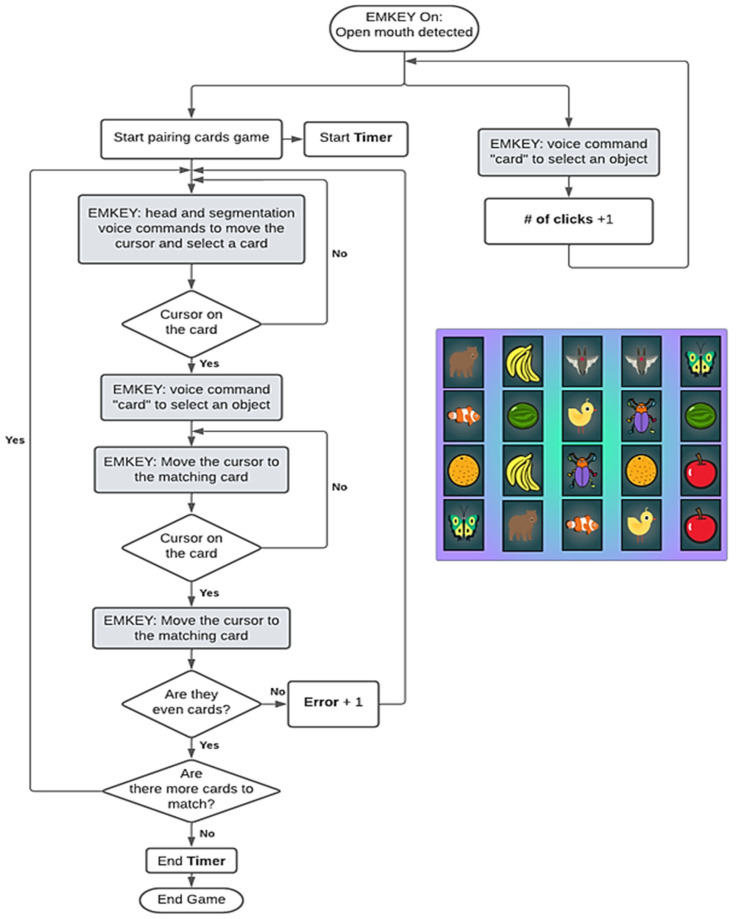
Pairing cards game flowchart and random array of figures to match.

This game was developed to test EMKEY’s cursor movement and segmentation commands. An eye-catching interface was developed with animation effects. At the start of the game, 10 pairs of fruit and animal figures were randomly displayed in a 5 × 4 array, as shown in Figure 3. The aim of the game was to match all card pairs in the shortest possible time. To select a card, the player had to pronounce the command ‘card’. For the purposes of this research, a click counter, an error counter and a timer were added to record data for each player.

#### 2.1.2. Maze Game

**Figure 4:**
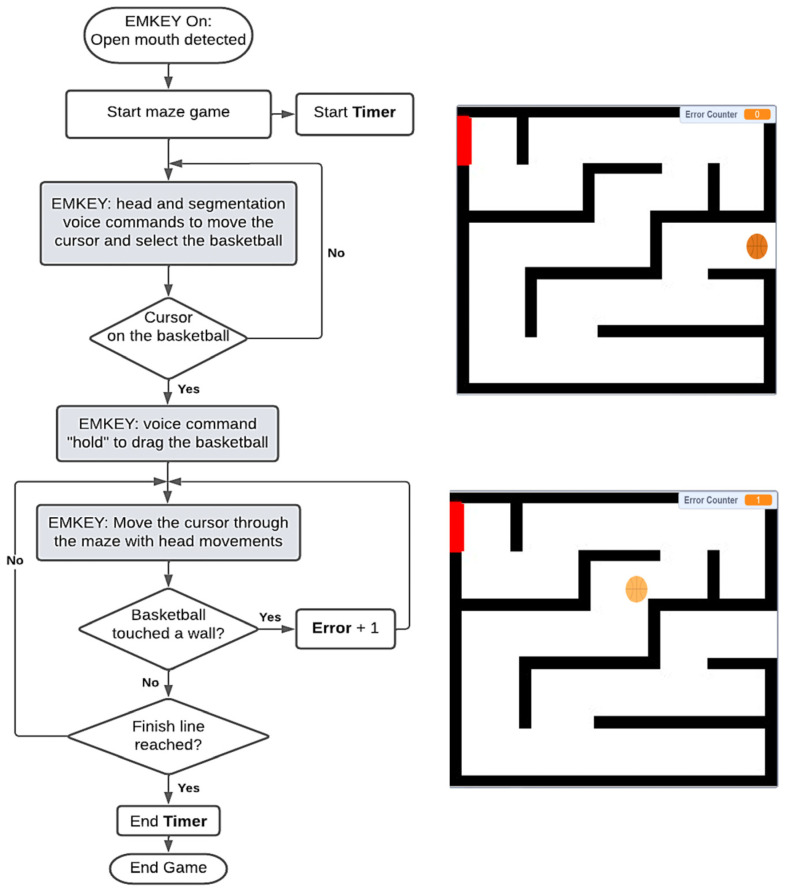
Maze game flowchart and interface. Finish line is the red rectangle in the maze.

This involved moving a basketball through a maze by holding down the mouse button and moving the cursor (Figure 4). The aim of the game was to reach the end line with the least number of falls. To start the game, the player had to hold the basketball and then move through the maze, avoiding collisions with the black walls. In this game, a timer and an error counter were added. Each time the basketball crashed, the error counter increased by 1. The players were not allowed to cross the walls and thus had to move all the way through. When playing with the emulator, the player had to move and touch the basketball with the cursor, and then pronounce the command ‘hold’ to activate the drag mouse function and move it through the maze.

#### 2.1.3. Car Crashing Experiment

**Figure 5:**
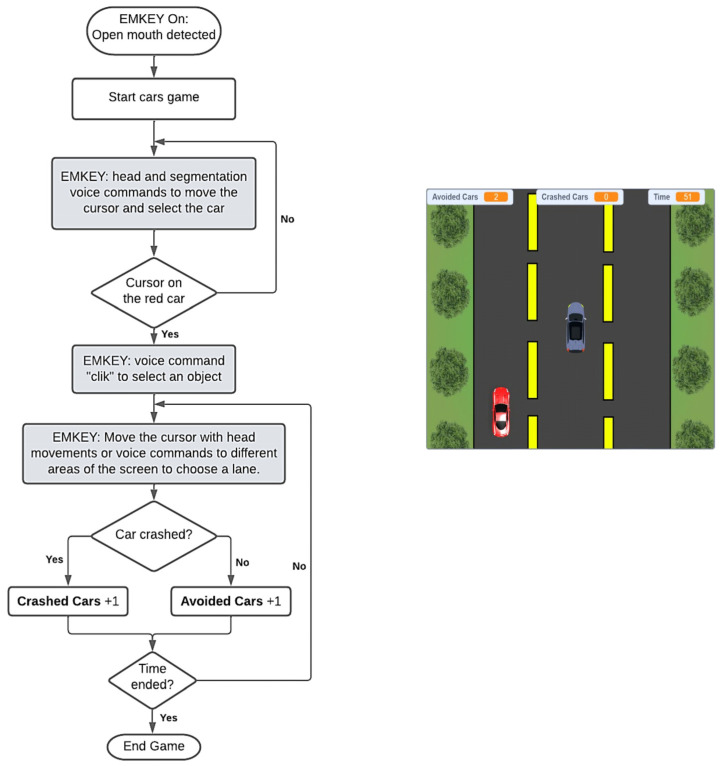
Car crashing game flowchart and interface.

This game involved controlling a car that moved to the right and left of a three-lane highway. An interactive scene (Figure 5) was developed to simulate a car moving on a road. Several cars appeared from above and the objective of the game was to avoid colliding with them. To control the car’s movement, the player had to move the cursor on one of the three paths of the road. In this case, a car-avoided counter and a car-crashed counter were added. Each player had to evade as many obstacles as possible within a period of 1 min.

For each game, we collected data from the variables implemented in each case, which were used to analyse EMKEY functionality. Table 1 shows the variables recorded for each game.

### 2.2. Tests

**Figure 6:**
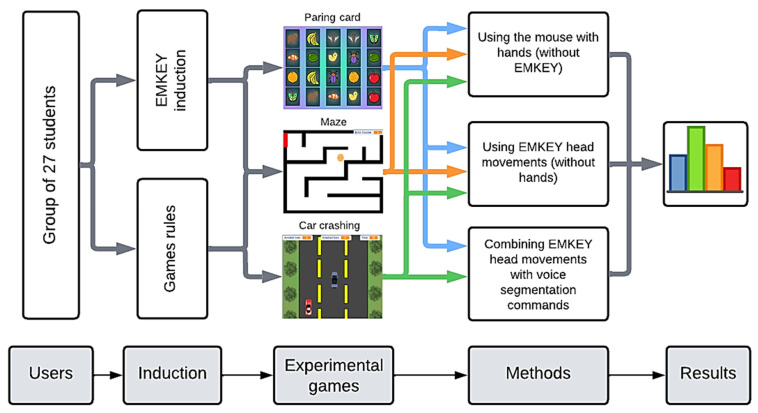
Process that the participants underwent.

The first step in testing EMKEY through the experimental games was an induction given to the study participants about the use of the EMKEY emulator and the aims of each game. The participants were informed about the main features of the voice commands and the correct way to generate cursor movement with nose tracking. A printed sheet of the PC screen segmentation was given to each user to help them to identify the most useful area to move to when needed. Each user played all three games under the different conditions listed in Table 2. A person experienced in the use of EMKEY and the operation of the games was responsible for conducting these evaluations and answering questions from users during the tests. A process diagram presented in Figure 6 represents the process that participants underwent for this study.

### 2.3. Research Hypothesis

In this study, the following seven hypotheses were proposed:

**Hypothesis** **1.** *There will be similar performances in terms of the number of clicks made by the participants when conducting the pairing cards experiment under the following three conditions: using the mouse with hands (without EMKEY), using EMKEY head movements (without hands) and combining EMKEY head movements with voice segmentation commands*.

**Hypothesis** **2.** *There will be similar times of execution of the pairing cards experiment under the following three conditions: using the mouse with hands (without EMKEY), using EMKEY head movements (without hands) and combining EMKEY head movements with voice segmentation commands*.

**Hypothesis** **3.** *There will be similar numbers of errors in the pairing cards experiment under the following three conditions: using the mouse with hands (without EMKEY), using EMKEY head movements (without hands) and combining EMKEY head movements with voice segmentation commands*.

**Hypothesis** **4.** *There will be similar times in carrying out the maze experiment under the following two conditions: using the mouse with hands (without EMKEY) and using EMKEY head movements (without hands)*.

**Hypothesis** **5.** *There will be similar numbers of errors when carrying out the maze experiment under the following two conditions: using the mouse with hands (without EMKEY) and using EMKEY head movements (without hands)*.

**Hypothesis** **6.** *There will be similar levels of obstacle avoidance in the car crashing experiment under the following three conditions: using the mouse with hands (without EMKEY), using EMKEY head movements (without hands) and combining EMKEY with the voice commands ‘two’, ‘centre’ and ‘three’ to move to the left, centre, and right lanes of the highway, respectively*.

**Hypothesis** **7.** *There will be similar numbers of crashed cars in the car crashing experiment under the following three conditions: using the mouse with hands (without EMKEY), using EMKEY head movements (without hands) and combining EMKEY with voice commands ‘two’, ‘centre’ and ‘three’ to move to the left, centre and right lanes, respectively*.

### 2.4. Participants

To calculate the sample size, a median effect size *η*^2^ = 0.30, alpha error probability α = 0.05 and a conventional statistical power of 1 − β = 0.80 were used as parameters. The results indicated 24 as an adequate sample size, which allowed for the selection of the study participants.

We worked with a sample of 27 participants. Regarding gender, 13 (48.1%) were female and 14 (51.9%) were male. The minimum age was 20 and the maximum was 24 years (*M* = 20.81, *SD* = 1.14). Regarding laterality, 25 (92.6%) were right-handed and 2 (7.4%) were left-handed. Regarding the hours of weekly computer use, the minimum was 2 h and the maximum was 20 h (*M* = 8.62, *SD* = 5.19).

The participants are university students from Quito, Ecuador. In all cases, they are in a healthy state and have normal upper extremities and motor performances. To carry out the experiments with the emulator, they did not use their arms, simulating the condition of a person with disabilities in the upper limbs. This decision was made in research to refine the EMKEY emulator before using it with people with a true disability.

The participants belonged to the careers of Psychology and Engineering. The socioeconomic stratum was medium and medium-high. In all cases, their voluntary participation was approved by signing an informed consent. There was good collaboration, since the ultimate goal of this study was to develop an emulator that allows people with upper limb disabilities to improve their living conditions.

Regarding the inclusion criteria in the study, it was that the participants had their extremities in perfect condition, in order to analyse the difference between the use of the emulator without the hands and the comparison of the performance when using the hands. No histories of addictions or neuropsychological disorders were noted. Regarding the age range, 20 and 25 years were established as parameters. Knowledge was required in the use of the computer and in the use of video games. The desire not to participate in the study and not meeting the previously mentioned criteria was used as the exclusion criteria.

The participants were recruited through non-probabilistic convenience sampling, by calling students from the university where the researchers of this study work. Once the sample size calculation necessary for the proposed study was made, the participants were selected.

### 2.5. Measurements

As previously mentioned, in this study, three experimental games were carried out: pairing cards, solving a maze and avoiding car crashing. For these tasks, 19 variables were measured:

V1: The number of clicks in the pairing cards game with the mouse and without EMKEY.

V2: The time for which the pairing cards game was run with the mouse and without EMKEY.

V3: The total errors in the pairing cards game with the mouse and without EMKEY.

V4: The number of clicks in the pairing cards game with EMKEY head movements.

V5: The time for which the pairing cards game was run with EMKEY head movement.

V6: The total errors in the pairing cards game with EMKEY head movements.

V7: The number of clicks in the pairing cards game with EMKEY head movements and voice segmentation commands.

V8: The time for which the pairing cards game was run with EMKEY head movements and voice segmentation commands.

V9: The total errors in the pairing cards game with EMKEY head movements and voice segmentation commands.

V10: The time for which the maze game was run with the mouse and without EMKEY.

V11: The total errors in the maze game with the mouse and without EMKEY.

V12: The time for which the maze game was run with EMKEY head movements.

V13: The total errors in the maze game with EMKEY head movements.

V14: The total evaded cars without EMKEY.

V15: The total crashed cars without EMKEY.

V16: The total evaded cars with EMKEY head movements.

V17: The total crashed cars with EMKEY head movements.

V18: The total evaded cars with EMKEY head movements and voice segmentation commands.

V19: The total crashed cars with EMKEY head movements and voice segmentation commands.

### 2.6. Data Analysis

We started with descriptive statistical calculations to characterise the measurements taken: mean, standard deviation, percentages, and minimum and maximum. Subsequently, to analyse the first three hypotheses, the ANOVA statistical procedure was used, considering the three conditions under which the experiment was carried out as a factor. In addition, Bonferroni’s post hoc correction was used to analyse the comparisons made. For the analysis of the remaining hypotheses, the mean comparison procedure was used for the two experimental conditions applied in the maze and car crashing experiments.

### 2.7. Procedure

The sample size was calculated based on a comparative experimental study under different conditions of emulator use. A pilot study was conducted to test the intervention strategy and to tune the emulator parameters to facilitate better playability. Then, the voluntary participation of young Ecuadorian university students who performed the experiments in a distraction-free environment was requested. Once the data were available, we performed the statistical analyses and wrote the research report. At all times during the investigation, the ethical standards of human subject research were maintained.

## 3. Results

### 3.1. Pilot Study: EMKEY Changes and Features

Three experienced EMKEY users tested the games, and some parameters were modified accordingly. First, to achieve a more comfortable control of the cursor, the speed of its movement was changed from 5 to 20 pixels/frame processed. In the first version of EMKEY [39], the speed was set to 5 because it is easier to select small icons on the screen, but the slow movement becomes stressful.

**Figure 7:**
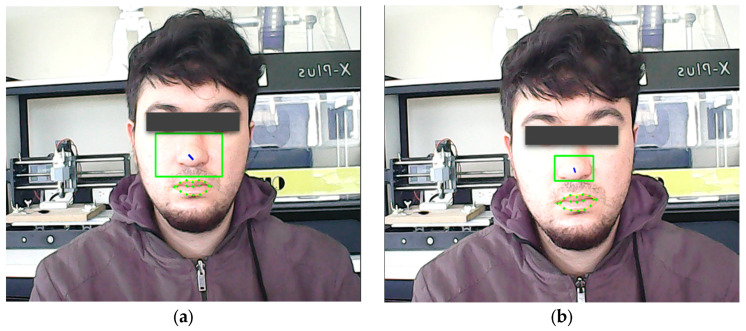
Green boundary box size comparison. (**a**) Rectangle of size 65 × 35 pixels/frame processed; (**b**) rectangle of size 35 × 20 pixels/frame processed.

The size of the green boundary box that appeared around the user’s face was also changed. In the first version, it was 65 × 35 pixels (Figure 7a). However, the user sometimes had to make uncomfortable neck movements to generate cursor movements; thus, it was changed to a smaller size of 35 × 20 pixels (Figure 7b). This area was used to define the boundaries that helped to generate cursor movements.

The EMKEY speech-to-text feature allows the user to emulate some mouse and keyboard functions. The only change in this aspect was the addition of some new commands, such as ‘select’ and ‘card’, for the pairing cards experiment.

The experiments were applied by the researchers of this study. It is important to note that any type of influence on the performance of the participants was controlled by using the same protocol for each of the participations. At no time was there any kind of help for any particular participant, or to favour the condition of the EMKEY emulator. In all cases, the three experimental conditions were applied in a place that was free of distractions and under the same conditions in all cases.

### 3.2. Results of Empirical Study Statistics

The statistical analysis was initiated using the descriptive values of the variables measured in the investigation. Table 3 shows the central tendency and dispersion data obtained.

In relation to the first hypothesis, no statistically significant differences were found when evaluating the number of clicks made by the participants in the pairing cards experiment (*F_(2,78)_* = 2.39, *p* = 0.10, *η*^2^ = 0.06). These data prove our hypothesis. Table 4 shows the values obtained for each experimental condition.

**Figure 8:**
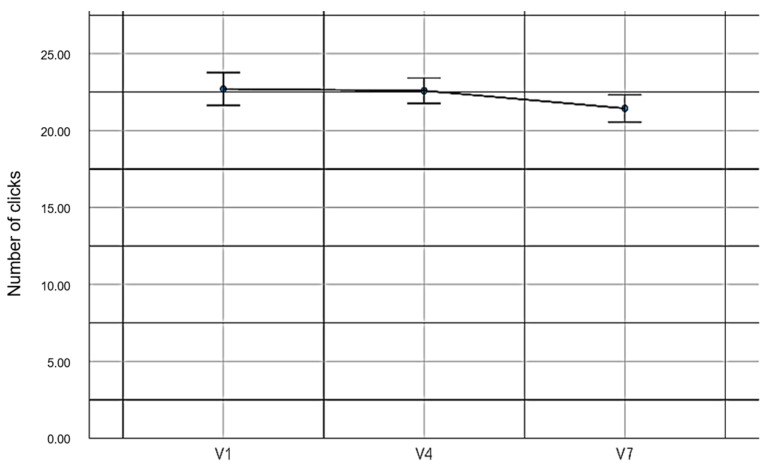
Number of clicks made in the pairing cards experiment.

Figure 8 shows graphically the number of clicks made in each condition of the pairing cards experiment.

Regarding the second hypothesis, statistically significant differences were found among the execution times in the pairing cards experiment under the three conditions executed (*F_(2,78)_* = 224.40, *p* ≤ 0.001, *η*^2^ = 0.85). These values do not support the proposed hypothesis. Table 5 shows the descriptive values of the time spent in the pairing cards experiment.

**Figure 9:**
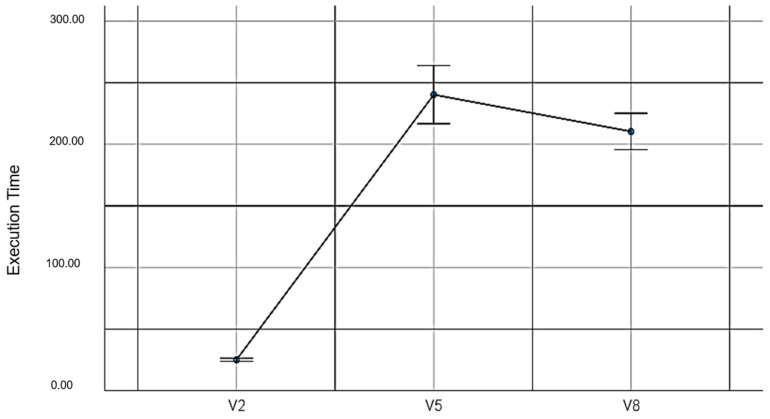
Execution times in the pairing cards experiment.

Figure 9 shows the execution times taken by the participants under the three conditions in the pairing cards experiment.

When comparing the numbers of errors in the pairing cards experiment, no statistically significant differences were found among the three experimental conditions (*F_(2,78)_* = 0.24, *p* = 0.79, *η*^2^ = 0.006). These results support the third hypothesis. Table 6 shows the descriptive values of the number of errors made in the experiment.

**Figure 10:**
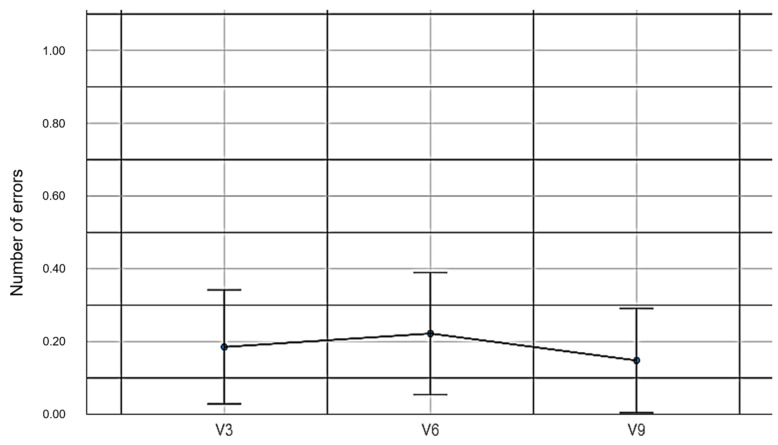
Numbers of errors made in the pairing cards experiment.

Figure 10 shows the numbers of errors made in the cards experiment under the three conditions.

When comparing the execution times in the maze experiment with the use of the mouse and the EMKEY emulator with head movements, statistically significant differences were found (*t_(52,1)_* = −18.45, *p* ≤ 0.001, *d* = 9.60). These data do not support the proposed hypothesis. Table 7 shows the values obtained in this comparison.

**Figure 11:**
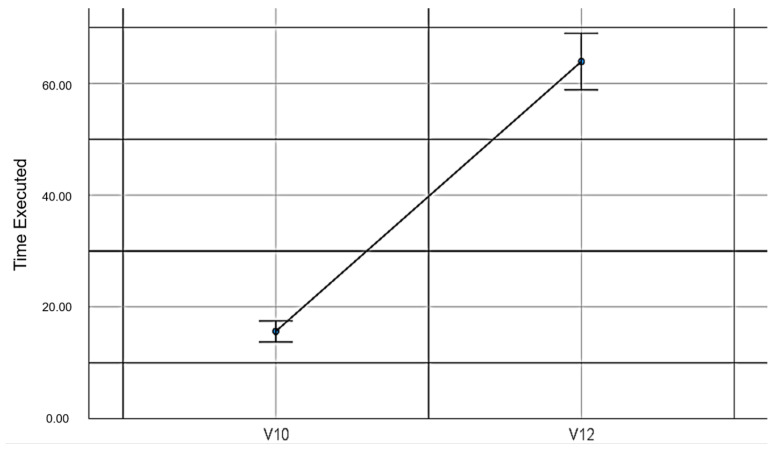
Execution times in the maze experiment.

Figure 11 graphically indicates the difference in the execution times in the maze experiment.

When comparing the number of errors made by the participants in the maze experiment with the use of the mouse and EMKEY with head movements, statistically significant differences were found (*t_(52,1)_* = −2.78, *p* = 0.004, *d* = 1.17). These findings do not support our fifth hypothesis. Table 8 shows the values obtained from this measurement.

**Figure 12:**
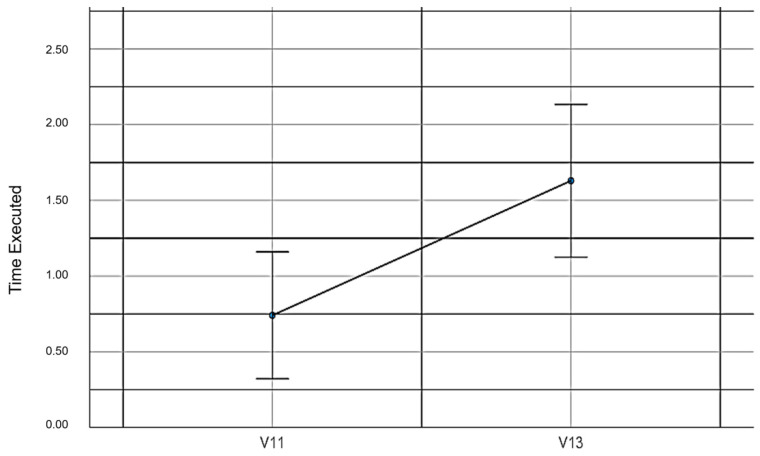
Errors made in the maze experiment.

Figure 12 compares the use of EMKEY and the mouse to conduct the maze experiment.

When evaluating the number of evaded obstacles in the car crashing experiment, statistically significant differences (*F_(2,78)_* = 85.33, *p* ≤ 0.001, *η*^2^ = 0.69) were obtained. These findings do not support our sixth hypothesis. Table 9 shows the descriptive values obtained in this experiment.

**Figure 13:**
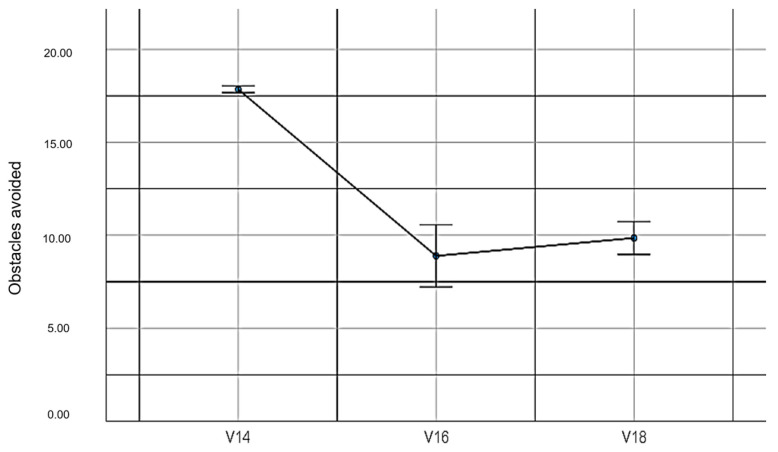
Number of obstacles evaded.

Figure 13 graphically represents the number of obstacles evaded under the three experimental conditions.

When evaluating the number of crashed cars under the three experimental conditions, statistically significant differences were found (*F_(2,78)_* = 71.26, *p* ≤ 0.001, *η*^2^ = 0.65). This finding does not support our seventh hypothesis. Table 10 presents the descriptive values of this comparison.

**Figure 14:**
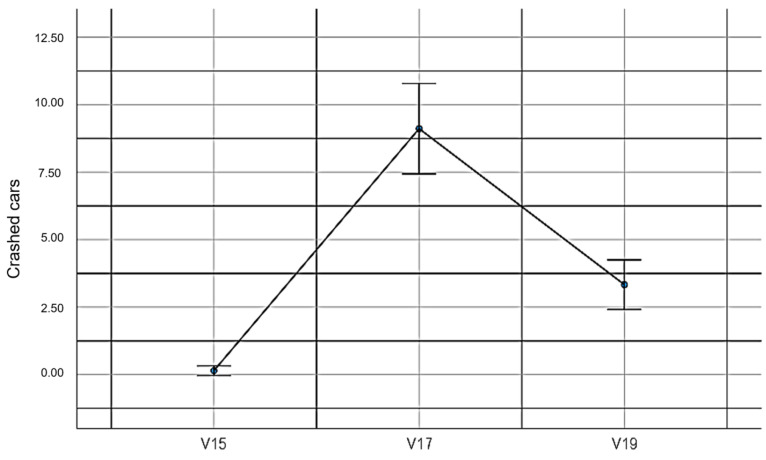
Number of crashed cars.

Figure 14 shows the values determined in the car crashing game under the three experimental conditions.

### 3.3. Summary Results

In this study, an experimental EMKEY platform testing was conducted. EMKEY is a machine interface that uses face and voice recognition algorithms to develop an assistive tool to help users to control a computer without a keyboard or mouse but only with head movements and some voice commands. It is a technological tool that allows people with upper limb motor disabilities to use a computer independently with all the advances that it represents.

Analysing the first hypothesis, no significant difference was found in the number of clicks to complete the game between the uses of EMKEY and a regular mouse. According to the results obtained in the three experiments, the emulator allowed the user to maintain total control to generate the click event, similar to the use of a common mouse. This is a positive result because it shows that the emulator does not generate phantom click events when the user does not give an appropriate command.

The results obtained for the second hypothesis showed a significant difference in the time taken by a user to complete the pairing cards game. The experimental results showed that the game could be completed faster using hands and a regular mouse than using EMKEY head movement. However, using the emulator with voice commands and head movements yielded better results than using head movements alone.

According to the results obtained for the third hypothesis, the numbers of errors in the pairing cards experiment with and without the use of EMKEY were similar. This shows that the main cause of errors in this experiment was not the emulator, which is favourable and demonstrates EMKEY reliability. Rather, the errors were attributable to the users or other external factors, such as anxiety and inexperience in handling the game.

According to the results obtained for the fourth hypothesis, the time taken by a user to carry out the maze experiment using EMKEY was greater than that taken using the hands with a mouse. These results are attributable to the functional characteristics of EMKEY, since the speed of the mouse cursor movement is predefined by the application, and its dragging options are limited to only one direction at a time, which restricts mobility. EMKEY delays partially originate from the preconfigured number of pixels that the mouse pointer moves when a head movement is detected; moreover, delays are introduced due to the recognition rate of the algorithm adopted. The time delay also varies according to the processing power of the PC.

In our fifth hypothesis, we found significant differences in the number of errors using EMKEY and the traditional mouse. This indicates the need to improve the precision of dragging stimuli on the screen. This function is extremely important because if we transfer the emulator to real computing environments, such as work or education, the person with motor disabilities must be able to correctly drag objects using EMKEY. This statement is related to previous research in which technological devices, such as EMKEY, have been applied to promote cognition in people with some type of cognitive or motor disabilities [47].

Regarding our sixth research hypothesis, there were more crashes when using EMKEY compared to using the mouse, which indicates the need to improve the sensitivity of the response and the mobility of the emulator for the benefit of people with motor disabilities. This advancement of the emulator will allow users to have more efficient mobility in the various computing tasks that need to be performed with our platform in the future.

Regarding the experiment of crashed cars in the seventh hypothesis, although EMKEY produced more crashed cars compared to the use of the same application without the emulator, when voice commands were used, the performance of the emulator improved.

## 4. Discussion

This study was developed with the aim of improving the usability level of EMKEY, focusing mainly on effectiveness and efficiency. Unlike other works that test similar interfaces with different data collection methods to measure the efficiency and effectiveness (Table 11); in our case, three experimental games were created using the Scratch programming language: card pairing, maze and car crash. These were used to test the main functions of EMKEY, such as moving the cursor on the screen, voice command recognition and mouse click events. A sample of 27 students participated in this study; 19 variables were analysed and 7 hypotheses were proposed to detect parameters that could help to improve the usability of a prototype.

As with previous related work that make human–computer interfaces for disabled people [11,12,13,14,15], the tests were conducted with non-disabled people as a first step due to the limited access to disabled participants. The results obtained allowed to identify usability characteristics that needed to be improved prior to the testing phase with people with disabilities.

According to the results obtained by hypothesis 1, 3, 5, 6 and 7, analysing the variables error and the number of clicks (see Table 1), EMKEY is an effective tool that allows users a hands-free control of the functions commonly commanded when using the mouse and keyboard of a computer. For evaluating the usability of our system, participants were trained to complete the proposed games. Speaking about aspects that evaluate the effectiveness, the authors in [14] developed a similar system that allows users with disabilities to interact with specific computer applications such as Google Chrome, Facebook and Gmail. This is performed by using head movements and specific voice commands. They also carried out a performance analysis of the voice commands to generate a click event. Their interface achieved a success rate of 76%, which means that the system had some problems in detecting a click. In our proposal, we measured the number of clicks a user had to make to complete a game. To calculate the success rate of the emulator’s voice commands, we used the data from the pairing cards test with head movements and voice commands. EMKEY’s success rate for a click event was 92.8%, which shows that EMKEY can perform speech recognition tasks with good performance. The tests related to the car crashing game measured the number of failures and successes in different scenes. When users used EMKEY voice commands, they obtained better results than just using their head to move the car. By using this functionality, it is proven that the emulator can be more useful and user-friendly for interacting with computers in comparison with the studies in [15,48] which are systems that control the use of the computer using only head movements and simple gestures. Implementing voice commands eliminates the need for users to make awkward facial gestures to emulate actions such as clicking.

In [49], a cursor control software was developed. The main feature of this system was that it could move the cursor fluidly across the screen using a predefined active region where a reference point is tracked and translated into a screen coordinate. This method to move the cursor makes the system more efficient. However, this proposal suffered from errors due to its high sensitivity, which generates less precision and accuracy. It prevents the selection of small objects on the screen. In contrast, a point in favour of EMKEY that was identified from the test games is that it allows a more precise selection of objects due to its speed and movements in one direction at a time, which proves its effectiveness.

To evaluate EMKEY efficiency, we consider hypotheses 2 and 4, through variable time (see Table 1). According to the results, the fluidity of cursor movement with EMKEY should be improved, since the time to complete specific tasks compared to the use of a regular mouse are significant. In this context, ref. [10] presents a hands-free computer interaction system for individuals with motor disabilities. To enable computer interaction, the system uses the Emotiv EPOC+ headset, which is an EEG device that captures signals from the user’s brain and translates them into commands to control the computer. The headset has 14 channels that capture signals from the brain, which are then processed to move the cursor on the computer screen. In addition, the headset has gyroscope and accelerometer sensors that track the user’s head movements to move the cursor in the corresponding direction. In contrast with our study, the authors make an efficiency analysis of their system by working with disabled and non-disabled people. Difficulty level and the time to complete a specific task were the analysed variables. The obtained results show that for people with motor impairments, the systems were easier to use but the users took longer to complete tasks compared with non-disabled users. In our case, users were subjected to two conditions: using a common mouse with the hand and using EMKEY to operate a computer. In this manner, we simulate users with and without upper limb motor dysfunctions to test our system. In the card and maze games, the difference between the time taken to complete each game using EMKEY and a normal mouse is longer, with the first one being the slowest. Extrapolating our results, we can say that disabled people will require more time to complete a task. This supports the fact that more consideration must be given to user adaptation factors such as the type of impairment, computer literacy, user experience, and so on. These considerations will contribute to improving the efficiency of our system.

From a clinical perspective, the development of the EMKEY emulator is a great contribution, since people with upper limb disabilities often have intact mental abilities; however, due to their disability, they lose their job or fail in their studies. Therefore, the development of this interface will have a positive impact on the quality of life of people with disabilities and their environment, since in the future they will be able to send emails, play video games, participate in video calls, fill in databases, etc. and earn a living by using computer skills. The results showed in this work are related to previous research, such as [50,51], which found that technological development positively impacts not only daily living activities, which is the objective of the study conducted with EMKEY, but also the brain functions of those who benefit from the technological device created.

As observed in the results of this investigation, EMKEY has some limitations. The main ones are focused on the aspect of efficiency. In the case of the cursor speed, it is low because it is intended to gain precision when selecting small objects on the screen. However, if the speed is higher, the effectiveness in this aspect decreases. In tasks that require the user to drag objects on the screen, it will be difficult to move them precisely. The limited time that the participant had to adapt to the system was also a problem in this study. Users may need some time to become proficient with assistive technology before using it, which can introduce variability in the experimental findings. Due to the parameters measured in the games, we have a limited number of variables to make a deeper analysis of the usability. However, this study is complementary to the previous work [39], where other parameters were measured such as user perception.

In the future, we will conduct research with improvements to the EMKEY emulator, for example, to implement some customisable options for controlling the cursor movement speed and adding some corrections to the drag and movement sensitivity. We will also add more variables and ways to acquire data that help in the usability analysis. In addition, we will continue conducting experimental studies with people with and without disabilities to support them with technological devices that assist them in their daily living activities. It must be taken into account that it is very difficult to find participants with motor disabilities and to expect them to be willing to participate in an experimental study. This limits the sample size.

## 5. Conclusions

The results of the comparative study performed through experimental games on the EMKEY platform evidenced the functionality of the emulator and identified specific features that should be improved to provide a more pleasant user experience. We verified that EMKEY allows for controlling mouse and click events effectively and without generating involuntary or ghost events. The voice and facial recognition functions facilitate the emulator operability. From the analysed hypotheses, it was identified that the emulator’s performance with respect to the speed of the cursor movement, as well as the precision of the mouse drag-and-drop function, needs to be optimised to improve its efficiency.

Note that the use of screen segmentation commands facilitated a reduction in the number of errors made by the user, as well as the execution time, under the conditions evaluated in the three experimental games. Thus, this function allows the user to control the movement of the mouse pointer more easily, somewhat compensating for the delays that occur in the mouse movement when only EMKEY’s head movement is used to move the pointer. The speed at which the mouse moves is a variable that can be tuned; however, the more sensitive it is, the more difficult it will be to select small objects on the screen. This may be a configurable parameter in future versions of EMKEY, depending on the user’s needs. The restricted mouse movement in only one direction at a time (vertical or horizontal) can also be improved in future versions to achieve (x, y) displacement. In addition, EMKEY’s performance will be affected by lighting conditions, as it will hinder facial recognition.

Finally, EMKEY is presented as a low-cost assistive technology tool that is aimed at helping people with upper limb disabilities. This approach aims to provide the end user with the best conditions for using a computer efficiently, as it is a tool to perform work, communicate and access information, which can improve their overall quality of life and increase their sense of independence and self-sufficiency.

## Figures and Tables

**Table 1:** Variables registered for each game.

Experiment	Time	Successes	Errors	# of Clicks
Pairing Cards	x	-	x (wrongly selected card)	x
Maze	x	-	x (wall crash)	-
Car Crashing	-	x (car avoided)	x (crashed car)	-

**Table 2:** Experimental conditions for each game.

Game	Condition 1	Condition 2	Condition 3
Pairing Cards	Using the mouse with hands (without EMKEY)	Using EMKEY head movements (without hands)	Combining EMKEY head movements with voice segmentation commands
Maze	Using the mouse with hands (without EMKEY)	Using EMKEY head movements (without hands)	-
Car crashing	Using the mouse with hands (without EMKEY)	Using EMKEY head movements (without hands)	Combining EMKEY head movements with voice commands ‘two’, ‘centre’ and ‘three’ to move to the left, centre and right lanes on the highway, respectively

**Table 3:** Description of the measurements performed.

Measurements	N	Minimum	Maximum	Mean	Std. Deviation
V1	27.00	20.00	29.00	22.70	2.67
V2	27.00	18.81	2432.00	114.25	463.22
V3	27.00	0.00	1.00	0.19	0.40
V4	27.00	20.00	28.00	22.59	2.08
V5	27.00	146.75	410.52	240.30	59.81
V6	27.00	0.00	1.00	0.22	0.42
V7	27.00	20.00	30.00	21.44	2.24
V8	27.00	141.74	27,991.00	1236.77	5347.00
V9	27.00	0.00	1.00	0.15	0.36
V10	27.00	10.03	1088.00	55.54	206.39
V11	27.00	0.00	4.00	0.74	1.06
V12	27.00	33.46	693.95	87.04	121.95
V13	27.00	0.00	4.00	1.63	1.28
V14	27.00	16.00	18.00	17.85	0.46
V15	27.00	0.00	2.00	0.15	0.46
V16	27.00	2.00	16.00	8.89	4.23
V17	27.00	2.00	16.00	9.11	4.23
V18	27.00	6.00	13.00	9.85	2.21
V19	27.00	0.00	7.00	3.33	2.32

Note: Variables V1 to V19 are described in Section 2.5.

**Table 4:** Descriptive values of clicks made in the pairing cards experiment.

Experimental Conditions	Mean	*SD*	Standard Error	Lower Limit	Upper Limit	Min	Max
V1	22.70	2.672	0.514	21.6	23.76	20.00	29.00
V4	22.59	2.080	0.400	21.76	23.41	20.00	28.00
V7	21.44	2.241	0.431	20.55	22.33	20.00	30.00

Note: Variables V1, V4 and V7 are described in Section 2.5.

**Table 5:** Time spent in the pairing cards experiment.

Experimental Conditions	Mean	*SD*	Standard Error	Lower Limit	Upper Limit	Min	Max
V2	25.07	3.07	0.59	23.86	26.29	18.81	30.98
V5	240.30	59.81	11.51	216.64	263.96	146.75	410.52
V8	210.43	37.24	7.17	195.70	225.16	141.74	279.91

Note: Variables V2, V5 and V8 are described in Section 2.5.

**Table 6:** Descriptive values of the number of errors made in the pairing cards experiment.

Experimental Conditions	Mean	*SD*	Standard Error	Lower Limit	Upper Limit	Min	Max
V3	0.19	0.40	0.08	0.03	0.34	0.00	1.00
V6	0.22	0.42	0.08	0.05	0.39	0.00	1.00
V9	0.15	0.36	0.07	0.00	0.29	0.00	1.00

Note: Variables V3, V6 and V9 are described in Section 2.5.

**Table 7:** Maze experiment runtimes.

Experimental Conditions	Mean	*SD*	Mean Standard Error
V10	15.64	4.75	0.91
V12	63.91	12.73	2.45

Note: Variables V10 and V12 are described in Section 2.5.

**Table 8:** Errors made in the maze experiment.

Experimental Conditions	Mean	*SD*	Mean Standard Error
V11	0.74	1.06	0.21
V13	1.63	1.28	0.25

Note: Variables V11 and V13 are described in Section 2.5.

**Table 9:** Obstacles avoided in the car experiment.

Experimental Conditions	Mean	*SD*	Standard Error	Lower Limit	Upper Limit	Min	Max
V14	17.85	0.46	0.09	17.67	18.03	16.00	18.00
V16	8.89	4.23	0.81	7.22	10.56	2.00	16.00
V18	9.85	2.21	0.43	8.98	10.73	6.00	13.00

Note: Variables V14, V16 and V18 are described in Section 2.5.

**Table 10:** Number of crashed cars.

Experimental Conditions	Mean	*SD*	Standard Error	Lower Limit	Upper Limit	Min	Max
V15	0.15	0.46	0.09	−0.03	0.33	0.00	2.00
V17	9.11	4.23	0.81	7.44	10.78	2.00	16.00
V19	3.33	2.32	0.45	2.42	4.25	0.00	7.00

Note: Variables V15, V17 and V19 are described in Section 2.5.

**Table 11:** Methodology used to acquire data for efficiency and effectiveness analysis.

Authors/Year	Type of Interface	Type of Users for Tests	Methodology Used to Acquire Data	Efficiency	Efficiency
(Šumak et al. 2019) [10]	Brain–computer interface using an EEG device	disabled/non-disabled	Various tasks such as sending an email, searching for an image in the gallery, downloading a book and reading a specific part of it, making a Skype call, finding a video on YouTube and shopping in online stores.	Yes	Yes
(Zhang et al. 2017) [11]	Eye tracking interface using a low-cost eye-tracker device	non-disabled	Through a searching task, they tested the system for a series of operations such as search, copy, paste, and so forth.	No	Yes
(Sias et al. 2017) [12]	Head tracking interface using gyroscope and accelerometers	non-disabled	Common tasks of pointing and selecting that are used in an interface with 13 circular targets, arranged in a circle in the centre of the screen.	Yes	Yes
(Mosquera et al. 2017) [13]	Face tracking interface using webcam	non-disabled	Common tasks of pointing and selecting that are used in an interface with 12 circular targets, arranged in a circle in the centre of the screen. Free navigation through the Facebook app	No	Yes
(Mosquera et al. 2020) [14]	Face tracking interface using webcam, combined with voice commands	disabled/non-disabled	Several tasks related to specific applications such as reading and sending emails in Gmail, performing a specific search and opening a link related to the topic in Google Chrome, searching for contacts in Facebook and browsing a profile and liking any post.	No	Yes
(Rahmaniar et al. 2022) [15]	Face tracking interface, eye landmarks detection with webcam	non-disabled	Software to test the system in which the user must move the cursor through several square boxes where they must click with horizontal movements of their head. The execution time, the accuracy for selecting objects and the efficiencies of the movements were measured.	Yes	Yes

## Data Availability

The data used to support the findings of this study are available from the corresponding author upon request.

## Abbreviations

The following abbreviations are used in this manuscript:

AT	Assistive technology
HCI	Human–Computer Interface
EEG	Electroencephalography
EMKEY	Emulator of Mouse and Keyboard
PC	Personal computer
*SD*	Standard deviation
